# Complete Genome Sequence of *Marinobacter* sp. CP1, Isolated from a Self-Regenerating Biocathode Biofilm

**DOI:** 10.1128/genomeA.01103-15

**Published:** 2015-09-24

**Authors:** Zheng Wang, Brian J. Eddie, Anthony P. Malanoski, W. Judson Hervey, Baochuan Lin, Sarah M. Strycharz-Glaven

**Affiliations:** a Center for Bio/Molecular Science and Engineering, Naval Research Laboratory, Washington, DC, USA; b American Society for Engineering Education, Washington, DC, USA

## Abstract

*Marinobacter* sp. CP1 was isolated from a self-regenerating and self-sustaining biocathode biofilm that can fix CO_2_ and generate electric current. We present the complete genome sequence of this strain, which consists of a circular 4.8-Mbp chromosome, to understand the mechanism of extracellular electron transfer in a microbial consortium.

## GENOME ANNOUNCEMENT

*Marinobacter* spp. are aerobic and moderately halophilic *Gammaproteobacteria* described as opportunitrophs with broad functional potential to adapt to and survive under diverse marine environments (e.g., seawater, the Arctic, hydrothermal plume, and petroleum field brines) (1), and are most well known for the ability to utilize hydrocarbons as their sole carbon and energy sources (2). Members of the genus *Marinobacter* have grown to include 36 validly described species, and 15 genomes have been published (1, 3–12). The majority of isolates come from investigations of hydrocarbon degradation and biogeochemical cycling. *Marinobacter* sp. CP1 was isolated from a self-regenerating and self-sustaining biocathode biofilm originally enriched from seawater collected at Rutgers, Marine Field Station (RUMFS) in Tuckerton, New Jersey, USA (13). The biocathode microbial community uses electrons supplied by a cathode to drive CO_2_ fixation and O_2_ reduction, and *Marinobacter* has been shown to be one of the most abundant constituents (13, 14). *Marinobacter* sp. CP1 alone can form a cathode biofilm resulting in extracellular electron transfer (EET) when supplemented with organic carbon; however, the maximum current was 2 orders of magnitude lower than that typically measured for the biocathode consortium. Genome analysis of *Marinobacter* sp. CP1 provides a reference to support ongoing multiomics studies characterizing the EET proteins and pathways of the biocathode consortium, as well as for determining the role of strain CP1 among other constituents, and ultimately improving biocathode performance.

The genome of *Marinobacter* sp. CP1 was sequenced by DNA Link USA, Inc. (San Diego, CA, USA) using the PacBio *RS* II sequencing platform (Pacific Biosciences, Menlo Park, CA, USA). Genomic DNA (14 µg) was extracted using the Wizard Genomic DNA purification kit (Promega) and used to prepare a 10-kb insert library that was sequenced using two single-molecule real-time (SMRT) sequencing cells and P4-C2 chemistry. This resulted in 8,205 filtered and preassembled sequence reads with a mean length of 7,314 bp and 105× genome coverage. Assembly (via SMRTpipe HGAP.2 and SMRTpipe Celera Assembler) and consensus polishing (SMRTpipe Quiver) yielded one linear contig with a size of 4,768,040 bp. A circular chromosome was obtained by filling a 382-bp gap between both ends of the contig with PCR. The size of the finished genome is 4,768,422 bp (56.93% GC content). The genome is predicted to contain 4,369 and 3,878 protein-coding sequences (CDS) using RAST (Rapid Annotation using Subsystem Technology) and the NCBI Prokaryotic Genome Annotation Pipeline, respectively, and contains 3 rRNA operons and 50 tRNAs. Two copies of the 16S rRNA gene differ from the third by 10 bp. Ribosomal RNA sequence heterogeneity within a bacterial genome is not uncommon (15) and is likely a result of horizontal gene transfer. The genome carries denitrifying reductase gene clusters nitrate reductase *narGHJI* and nitrous oxide reductase *nosLYFDZR*, suggesting that this bacterium is able to use nitrate as an electron acceptor and eliminate nitrosative stress caused by nitrous oxide under microaerobic or anaerobic conditions within the biofilm (16).

### Nucleotide sequence accession number.

The complete genome sequence of *Marinobacter* sp. CP1 has been deposited in GenBank under accession number CP011929.
